# PHYLOGENETIC POSITION OF *ZYGOGONIUM ERICETORUM* (ZYGNEMATOPHYCEAE, CHAROPHYTA) FROM A HIGH ALPINE HABITAT AND ULTRASTRUCTURAL CHARACTERIZATION OF UNUSUAL APLANOSPORES

**DOI:** 10.1111/jpy.12229

**Published:** 2014-10-01

**Authors:** Rosalina Stancheva, John D. Hall, Klaus Herburger, Louise A. Lewis, Richard M. McCourt, Robert G. Sheath, Andreas Holzinger

**Affiliations:** Department of Biological Sciences, California State University San Marcos, San Marcos, California 92096, USA; Department of Plant Science and Landscape Architecture, University of Maryland, College Park, Maryland 20742, USA, Department of Botany, Academy of Natural Sciences, Philadelphia, Pennsylvania 19103, USA; Institute of Botany, University of Innsbruck, Sternwartestraße 15, Innsbruck A-6020, Austria; Department of Ecology and Evolutionary Biology, University of Connecticut, Storrs, Connecticut 06269, USA; Department of Botany, Academy of Natural Sciences, Philadelphia, Pennsylvania 19103, USA, Department of Biodiversity, Earth, and Environmental Sciences, Drexel University, Philadelphia, Pennsylvania 19104, USA; Department of Biological Sciences, California State University San Marcos, San Marcos, California 92096, USA; Institute of Botany, University of Innsbruck, Sternwartestraße 15, Innsbruck A-6020, Austria

**Keywords:** alpine habitat, generic and subgeneric concept, green algae, morphology, phylogeny, reproduction, *Zygnema*, Zygnematophyceae, *Zygogonium*

## Abstract

*Zygogonium ericetorum*, the type species of the genus, was studied from a natural population collected in Mt. Schönwieskopf, Tyrol, Austria. Generic concepts of *Zygogonium* and *Zygnema* were tested with *atpB, psbC*, and *rbcL* gene sequence analysis, which showed a sister relationship between *Z. ericetorum* and *Mesotaenium*, in an early branching clade sister to a grouping of *Zygnema* and several other filamentous and unicellular zygnematalean taxa. A variety of light, confocal, transmission electron microscopy, and cytochemical techniques provided new data on the variable chloroplast shape of *Z. ericetorum*, and its aplanospore structure and development, which has been previously considered taxonomically important but has been ambiguously interpreted. *Zygogonium* can be distinguished from other zygnematophytes (particularly *Zygnema*), based on the combination of two characters: (i) irregular, compressed plate-like chloroplasts and (ii) residual cytoplasmic content left in sporangia outside of the fully developed aplanospores or zygospores. The presence of a sporangial wall that separates the spores from the parent cell should be excluded from the definition of *Zygogonium*, because it is also observed in *Zygnema*. Similarly, the ecological characterization of *Zygogonium* as acidophilic is not unique to the genus. The names of 18 species currently belonging to *Zygogonium* are here changed to *Zygnema*, because of incompatibility with this new proposed *Zygogonium* concept. In the species transferred to *Zygnema*, chloroplasts are typically stellate in three-dimensions, and the entire content of fertile cells is transformed into the spore, so there is no cytoplasmic residue.

The enigmatic green algal genus *Zygogonium* Kützing and its type species, *Zygogonium ericetorum*, have confounded phycologists for decades with their peculiar vegetative and reproductive morphology. Czurda (1932) considered *Zygogonium* and *Zygnema* C. Agardh synonymous because of vegetative similarities. Transeau (1933) amended *Zygogonium* based on three characteristics: (i) vegetative cells with a pair of rounded, plate-like or irregular pillow-shaped chloroplasts, each with a central pyrenoid; (ii) formation of a sporangium wall which divides the gametangium before the formation of the spore wall, and (iii) presence of cytoplasmic residue in the mature gametangia. Despite the clear delineation of chloroplast shape by Transeau (1933), this important taxonomic feature has been subsequently described for some *Zygogonium* species in a questionable fashion. For instance, in *Zygogonium tunetanum* Gauthier-Lièvre the chloroplasts have been reported as irregularly spherical or stellate (Gauthier-Lièvre 1965, Wei et al. 1989, Wei and Yung 2000). Some authors considered both genera *Zygnema* and *Zygogonium* to have stellate chloroplasts (Kadlubowska 1984) and thus to be closely related phylogenetically (Gontcharov et al. 2003). A large number of species of *Zygogonium* with variable vegetative characteristics and ecological preferences have been reported (e.g., Randhawa 1959, Kadlubowska 1984).

Stancheva et al. (2012) showed that some *Zygnema* species also possess a combination of reproductive and vegetative features noted as characteristic of *Zygogonium* by many authors (e.g., Transeau 1951, Gauthier-Lièvre 1965, Kadlubowska 1984, Rundina 1998), such as zygospores formed in the conjugation tubes and separated from the gametangia by a cellulosic sporangial wall, predominant asexual reproduction by aplanospores and akinetes, presence of short branches, single-celled or filamentous rhizoidal outgrowths, thickened vegetative cell walls, purple-colored cell content, and transverse walls. These observations confound the existing generic classification systems of Zygnemataceae, which are based on the shape of chloroplasts and structure of fertile cells (for historical review see Yamagishi 1963). Molecular phylogenetic investigations determined that *Zygogonium* was sister to a clade of *Zygnema* spp. based on the only available strain of *Z. tunetanum*, in contrast to *Zygnemopsis*, which was distantly related to *Zygnema* (Hall et al. 2008, Stancheva et al. 2012). Stancheva et al. (2012) concluded that many species of *Zygogonium* are probably best treated as *Zygnema* and described two new species with many vegetative and reproductive characteristics of *Zygogonium*, except for the presence of cytoplasmic residue as *Zygnema*. However, the phylogenetic position of genus *Zygogonium* sensu stricto and its existence as a taxonomic entity distinct from *Zygnema*, remained unclear until the type species (*Z. ericetorum*) could be confidently identified and thoroughly studied.

Because of the known difficulties in distinguishing between *Zygnema* and *Zygogonium*, we will limit further discussion of systematics and ecology of *Zygogonium* to those few well-documented records that are entirely consistent with that of the type species. *Z. ericetorum* is a cosmopolitan species and based on the few confirmed sites, occasionally forms large single-species communities in acid pools, sphagnum peat bogs and on wet acid soils (Transeau 1933). Its ability to withstand desiccation in terrestrial habitats has been known for a long time (Fritsch 1916, Fritsch and Haines 1923), and this is an ecological ability has been observed in other representatives of Zygnematophyceae (for summary see Holzinger and Karsten 2013, Karsten and Holzinger 2014). However, many records of *Z. ericetorum* are based on vegetative filaments and the mostly acidic habitat in which the organism was distributed (e.g., Lynn and Brock 1969, Hoppert et al. 2004, Kleeberg et al. 2006, Newsome and van Breemen 2012), and neither of these characters is diagnostic (Stancheva et al. 2012).

Holzinger et al. (2010) and Aigner et al. (2013) recorded *Z. ericetorum* in high-alpine ephemeral streamlets with slightly acidic water in the Alps and provided electron microscopy visualization of vegetative cells along with ecophysiological data. *Zygogonium ericetorum* was demonstrated to be desiccation-tolerant in the vegetative state in field samples, showing intact cell organelles in fully desiccated samples (Holzinger et al. 2010). Aigner et al. (2013) reported substantially elevated levels of soluble compounds, such as phenolics and hydrolysable tannins, in purple-colored filaments of *Z. ericetorum* isolated mainly from the partially desiccated top layers of the mat, compared to the green filaments from lower layers.

Despite the interesting ecophysiological and ultrastructural features described, no phylogenetic characterization of *Z. ericetorum* has been performed to date, and such an analysis is critical to determine the placement of filaments attributed to this genus (Stancheva et al. 2012). Therefore, we conducted this investigation of *Z. ericetorum*, using fresh living material from the Alps to (i) evaluate its phylogenetic placement within Zygnematophyceae and (ii) determine vegetative and reproductive characters relevant to generic concepts of *Zygogonium* and *Zygnema*.

## Materials and Methods

### Algal material

This study was conducted on a natural population of *Z. ericetorum* collected from Mt. Schönwieskopf (46°50′ 998 N, 11°00′ 903 E), at 2,350 m a.s.l. near Obergurgl, Tyrol, Austria as previously reported (Holzinger et al. 2010, Aigner et al. 2013). Climate and microclimate data for the collection site are already available (Karsten et al. 2010). *Zygogonium ericetorum* samples were collected by hand from four different areas in a spring pool on July 18, 2013 and kept at moderate temperature until processed in the laboratory in Innsbruck for confocal laser scanning microscopy (CLSM) and fixation for transmission electron microscopy (TEM; see below). *Zygogonium ericetorum* filaments were incubated in water from the habitat and placed in plastic petri dishes with Bold’s Basal Medium (BBM; Nichols and Bold 1965) with 1.5% agarose added (hard BBM).

Two samples were transported to the laboratory at California State University San Marcos, California, USA within 5 d of their collection, with light microscopic observations made directly on fresh field material and from material grown in culture as described below. Two macroscopically distinguishable morphs of filaments were collected from mats: purpletinted filaments from the surface layers, which were exposed to high sun light and desiccation, and green filaments from the lower layers, which received less radiation and contained more moisture. Each filament morph was cultured in both liquid BBM and 2% agar BBM, latter sprayed every 3 d with distilled water, both on 12:12 light:dark cycle at 12°C (Andersen 2005). The filaments were checked every 3 d up to 35 d and different stages of filament and aplanospore development under various media conditions were documented by microphotographs from living material.

### Phylogenetic analysis

Filaments of *Z. ericetorum* were isolated using forceps for DNA extraction. Two extractions were made from the field sample described above (strains JH1396 and JH1397). The DNA was extracted using the Nucleon Phytopure DNA extraction kit (GE Healthcare, Pittsburgh, PA, USA), as previously described (Hall et al. 2008). Portions of the chloroplast genes *atpB, psbC,* and *rbcL* were amplified using published primers and protocols (Hall et al. 2008, Stancheva et al. 2013, Peréz et al. 2014). Attempts to amplify other commonly used loci from the samples were unsuccessful (not shown). DNA sequencing and phylogenetic analyses were performed as previously reported (Stancheva et al. 2013). Briefly, maximum parsimony (MP) analyses were performed in PAUP* (Swofford 2003) with 10 random additional sequences. Bootstrap support was estimated using 500 bootstrap replicates. Maximum likelihood (ML) analyses were performed using RAxML (Stamatakis et al. 2005, 2008) via the Cipres Science Gateway (Miller et al. 2010). Analyses using Bayesian inference (BI) (Ronquist and Huelsenbeck 2003) were also performed via Cipres (Miller et al. 2010) using two chains run for 10 million generations with the first quarter of the trees discarded as burnin. Loci were analyzed individually and in combined analyses with all three loci. In addition to these analyses, partitioned data sets were also analyzed using MrBayes v. 3.2.2 (Ronquist and Huelsenbeck 2003); each locus partitioned by codon position and a combined (3 locus) data set partitioned by locus. Finally, a supplementary analysis of *rbcL* with three additional sequences distantly related to *Z. ericetorum* (two *Mesotaenium* strains as well as *Fottea pyrenoidosa* Broady) was performed following the methods described above and presented in Figure S1 in the Supporting Information.

### Light and confocal microscopy

An Olympus BX41 light microscope and Olympus IX50 inverted fluorescence microscope with an attached Olympus MicroFire S99809 digital camera (Olympus Imaging America Inc., Center Valley, PA, USA) were used for specimen observation and microphotography. The size data given in the descriptions are based on a minimum of 20 specimens measured and analyzed using Rincon image analysis software (Imaging Planet, Goleta, CA, USA). The chemical composition of the cell wall was examined using cytochemical techniques for the localization of pectic substances (Ruthenium Red, Jensen 1962) and cellulose (Calcofluor White [CFW], Krishnamurthy 1999), using chemicals from Fisher Scientific, Pittsburg, PA, USA. CFW selectively binds to cellulose and chitin. The dye fluoresces when exposed to UV light (340–380 nm) and offers a very sensitive method for direct microscopic examination of cell wall content and structure. For chlorophyll autofluorescence, filaments were excited with FITC filter (excitation: 467-498 nm, emission: 513–556 nm, Olympus Scientific Solutions Americas in Waltham, MA, USA).

CLSM was performed with a Zeiss Pascal 5 on a Zeiss Axiovert 200 M microscope (Carl Zeiss, Jena, Germany), equipped with a 63×1.4 NA objective lens. An argon laser was used at 488 nm in single track mode (21% laser power, main dichroic beam splitter a HFT 488, 93 μm pinhole), for excitation of the chloroplast autofluorescence and the emitted signal was collected with a 560-nm long pass filter and false colored red. In parallel, a bright field image was collected at channel D with a transmission photomultiplier tube. The two images were either merged or only the chloroplast autofluorescence is shown here.

### Transmission electron microscopy

TEM was performed with field-collected samples according to the methods described by Holzinger et al. (2010). Briefly, field samples were fixed for 1.5 h in 10 mM sodium cacodylate buffer, pH 6.8, containing 1.25% glutaraldehyde. After several rinsing steps, samples were postfixed over night in 1% OsO_4_ at 4°C, dehydrated in increasing ethanol concentrations and transferred to modified Spurr’s resin (Ellis 2006) via propylene oxide (2:1 propyleneoxide:Suprr’s resin, 1:2 propylenoxide: Spurr’s resin), for 2.5 h each, continuing by an overnight infiltration step in pure Spurr’s resin. Samples were then heat polymerized at 80°C for 8 h, sectioned on a Leica ultracut, counterstained with uranyl acetate and Reynold’s lead citrate. Sections were examined by a Zeiss LIBRA 120 TEM at 80 kV. A Proscan 2 k SSCCD camera (Proscan Electronic Systems, Lagerlechfeld, Germany) was used for image generation. Images were processed with Adobe Photoshop 7.0 software (Adobe Systems Inc., San José, CA, USA).

## Results

### *Zygogonium ericetorum* phylogeny

Zygogonium ericetorum *phylogeny*. Using PCR, we were only able to obtain one sequence of *atpB* from extraction JH1396 (Table S1 in the Supporting Information). We obtained identical *psbC* sequences from two different extractions. We also obtained identical sequences of *rbcL* from two different extractions, although one was only a partial sequence. These two sequences were highly similar to an *rbcL* sequence obtained by coauthor Lewis from a previous, unrelated extraction (data not shown). Data sets for these three chloroplast loci containing a large number of conjugating green algae (Zygnematophyceae) and *Coleochaete* (as an outgroup) were assembled. The *atpB* data set included 62 taxa and 1,207 nt characters of which 544 characters were variable and 504 characters were parsimony informative. The *psbC* data set included 38 taxa and 1,202 nt of which 518 were variable and 468 were parsimony informative. The *rbcL* data set included 62 taxa and 1,335 nt of which 567 were variable and 512 were parsimony informative. The combined, 3-locus data set contained 3,744 characters of which 1,629 characters were variable and 1,482 characters were parsimony informative.

Each locus was aligned and analyzed independently. In all analyses, *Z. ericetorum* was sister to *Mesotaenium* sp. JH0031 (or a clade of *Mesotaenium* sp. JH0031, *Mesotaenium* sp. AG2009-1 and *F. pyrenoidosa* in the supplementary analysis of *rbcL* (Fig. S1) with variable moderate to high levels of statistical support depending on the method. No strains of conjugating green algae were closely related to *Z. ericetorum* compared to genus-level relationships in other clades (e.g., within Desmidiales).

In the supplementary analysis of *rbcL*, the clade of *Z. ericetorum, Mesotaenium* sp. JH0031, *Mesotaenium* sp. AG2009-1, and *F. pyrenoidosa* was embedded in a clade containing *Mesotaenium* cf. *chlamydosporum* (Fig. S1). This clade received high statistical support, but its placement in the overall phylogeny of conjugating green algae was not strongly supported. Because of missing data in these *Mesotaenium* and *Fottea* strains and because no strain was more closely related to *Z. ericetorum* than *Mesotaenium* sp. JH0031, further results and discussion will be limited to the core data sets.

**Fig. 1:**
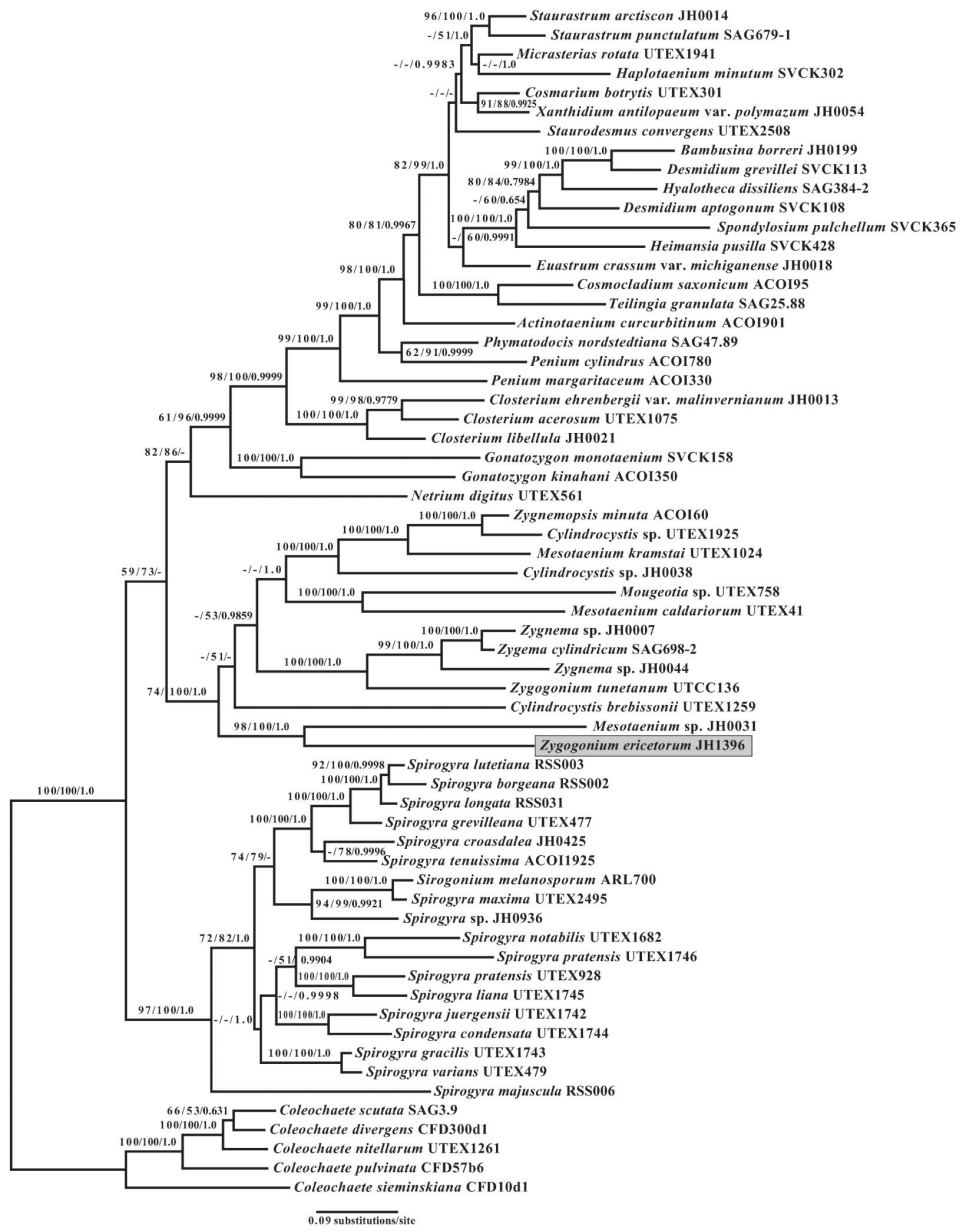
Phylogeny of Zygnematophyceae based on a multigene RAxML analysis of the chloroplast genes *atpB*, *psbC* and *rbcL* analyzed as a single partition. *Zygogonium ericetorum* is highlighted in gray. Numbers above the branches represent bootstrap values from maximum parsimony and RAxML analyses and posterior probabilities from Bayesian inference, respectively. Bootstrap values less than 50 and posterior probabilities less than 0.5 are indicated with a dash.

Placement for the *Z. ericetorum* and *Mesotaenium* sp. JH0031 clade varied considerably among analyses with respect to the branching order of other early diverging zygnematophytes. Additionally, statistical support for the placement of the early diverging clades was very low in most analyses of most loci (data not shown). Because there was no statistically strong disagreement among loci in bootstrap analyses of single locus data sets, these loci were combined into a 3-locus data set that was analyzed as one large partition as well as with three partitions corresponding to each gene. In the single-partition 3-locus data set, *Z. ericetorum* was sister to *Mesotaenium* sp. JH0031 with high statistical support (98% bootstrap support in Parsimony analysis, 100% bootstrap support in RAxML Maximum Likelihood analysis and 1.0 posterior probability in BI; Fig. 1). Inclusion of these taxa in a larger clade containing all other Zygnematales except *Spirogyra* and *Netrium digitus* (Brébisson ex Ralfs) Itzigsohn and Rothe was strongly supported in likelihood analyses (100, 1.0), but poorly supported (74) in parsimony analyses (Fig. 1).

Sister relationships between the *Z. ericetorum* + *Mesotaenium* sp. JH0031 clade and other Zygnematales varied among analyses. In the MP analysis, the *Z. ericetorum* + *Mesotaenium* sp. JH0031 clade was observed to be sister to a clade containing *Zygnemopsis, Zygnema* spp. (including the dubious *Zygogonium tunetanum*), *Mesotaenium kramstai* Lemmermann, and most *Cylindrocystis* spp. In the BI analyses of the combined data set, the *Z. ericetorum* + *Mesotaenium* sp. JH0031 clade was found in a polytomy with *Cylindrocystis brebissonii* (Ralfs) DeBary UTEX1259 and a clade containing most Zygnematales (except *Spirogyra* and *N. digitus*) (not shown). ML analysis of the 3-locus data set in RAxML found the *Z. ericetorum* + *Mesotaenium* sp. JH0031 clade sister to all other Zygnematales (excluding *Spirogyra* and *N. digitus*; Fig. 1). Support for the inclusion of *Z. ericetorum* was high (100), but the placement of the *Z. ericetorum* + *Mesotaenium* sp. JH0031 clade with respect to other lineages in the Zygnematales remained poorly supported (Fig. 1).

The topology of the 3-locus data set partitioned by locus found using BI was the same as the topology from the RAxML analysis of the combined data set (data not shown, cf. Fig. 1), although statistical support and branch length differed. Similarly, there was strong support for the sister relationship between *Z. ericetorum* and *Mesotaenium* sp. JH0031 (1.0), and strong support for their inclusion in the main clade of Zygnematales (1.0), but weak support for relationships among deeper clades of the Zygnematales (data not shown). In all analyses of all data sets, *Z. ericetorum* was sister with *Mesotaenium* sp. JH0031 and outside of the main clade of *Zygnema* spp. (including *Z. tunetanum*).

### Light and confocal microscopy of the vegetative and reproductive morphology of *Z. ericetorum.* Filament morphology

**Fig. 2:**
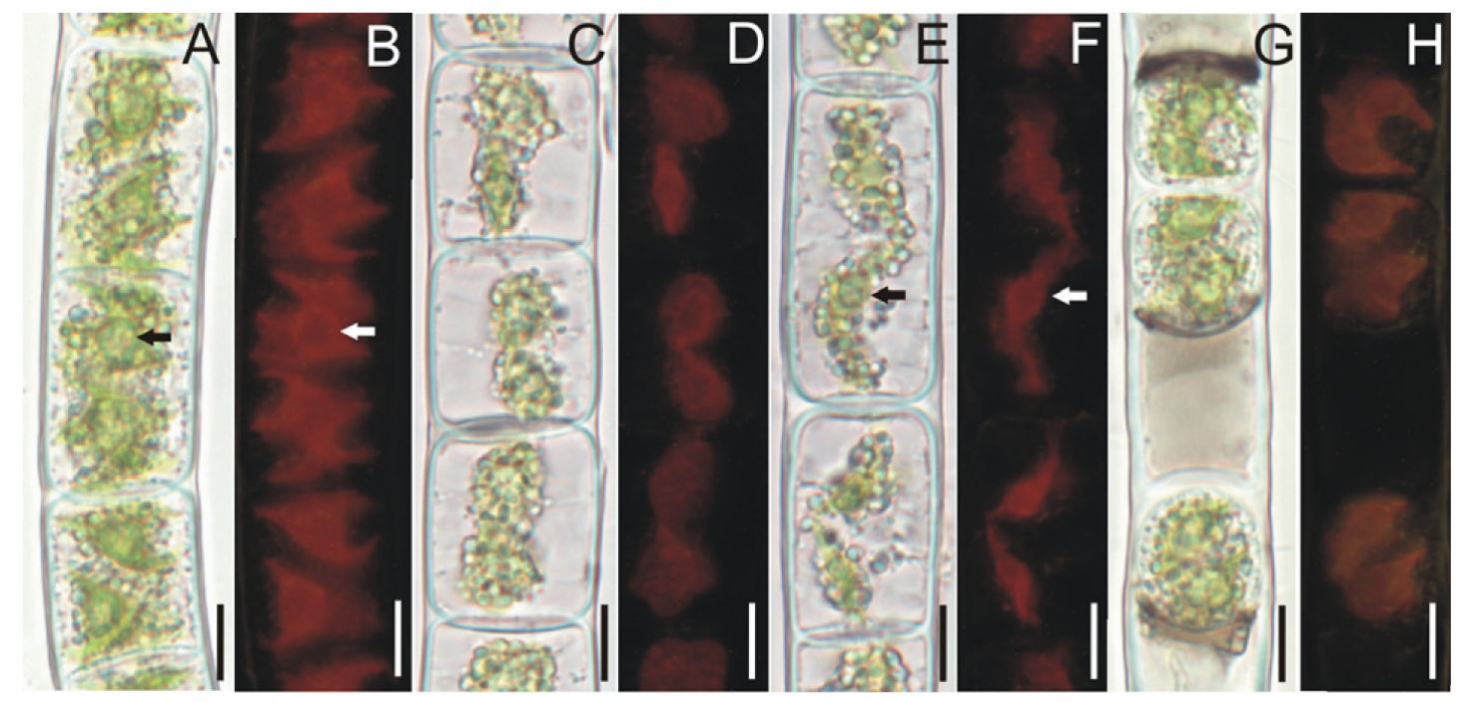
Light microscopic (A, C, E, G) and fluorescence (B, D, F, H) images of chloroplasts in *Zygogonium ericetorum*: (A, B) short vegetative cells with large deep green chloroplasts each with three to five protrusions, (C, D) short vegetative cells with light purple cell content and small rounded plate-like chloroplasts, (E, F) long vegetative cells with light purple cell content and longitudinally elongated plate- or ribbon-like chloroplasts, (G, H) cells with aplanospores each with two chloroplasts. Arrows show pyrenoids. Scale bars: 10 μm.

Filaments were unbranched or occasionally with short branches, and unicellular or multicellular rhizoidal outgrowths. Vegetative cells were cylindrical, 15–31 μm wide, 10–123 μm long. The cell wall was two-layered with the inner layer cellulosic, and outer layer pectic. Filaments of the green morph, grown in liquid BBM, had short cells, large, deep green chloroplasts, thin cell walls and colorless cell content (Fig. 2A). When grown on agar, green filaments formed thicker, H-shaped pieces in the pectic layer of the cell wall and often dissociated into short, few-celled segments with rounded cell ends; cells were occasionally slightly inflated. Filaments of the purple morph, grown on agar, had more elongate cells with pinkish colored cell content (Fig. 2E), proportionally smaller light green chloroplasts, thicker multilayered cell walls with pectic material deposited between the cellulosic layers, and frequent H-shaped wall structures. Filaments with purple cell content, directly observed from the field material, formed numerous aplanospores (Figs. 2G; 4, A–C; and 5, described below). By contrast, akinetes were rarely observed. Akinetes were cylindricalovoid to oblong, 19–25 μm wide, 28-52 μm long, completely filling the cell, and were covered by thick multilayered colorless wall (Fig. 4D). Zygospore formation has not been observed.

### Chloroplasts

**Fig. 3:**
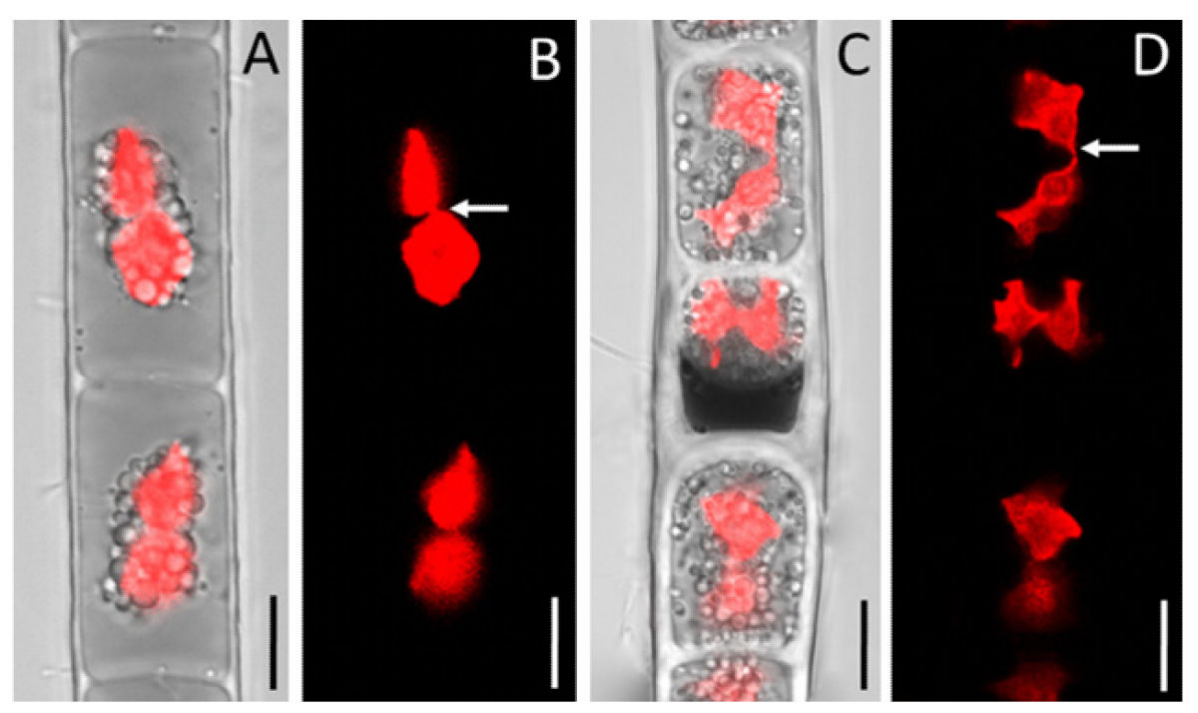
Confocal laser scanning microscopic images of chloroplasts in *Zygogonium ericetorum:* (A, B) cells with small rounded plate-like chloroplasts, (C, D) cells with irregular plate-like chloroplasts and short protrusions. Note the diagonal position of chloroplasts in each cell which displaced their wide front face and narrow profile. Arrows show the contact area between two chloroplast. Scale bars: 10 μm.

Each cell contained two chloroplasts with highly variable morphology (Figs. 2; 3; and S2 in the Supporting Information). In the shorter, actively dividing cells of green filaments the two chloroplasts almost completely covered the cell circumference (Fig. 2A). Each chloroplast appeared as an irregularly rounded plate with two to five, typically four, irregular peripheral protrusions in one plane and a massive, swollen, pillow-shaped central region holding a large, and distinct pyrenoid (Fig. 2, A, B, E, and F). Both chloroplasts were bent against the walls and positioned opposite to each other with their massive central regions facing the cell walls and protrusions folded toward the middle of the cell. In the longer cells with purple cell contents, the chloroplasts occupied only the central portion of the cell and were obscured by the presence of lipid droplets and starch grains. The chloroplasts were more rounded (Figs. 2, C and D and 3, A and B) or taeniform (Fig. 2, E and F) with very short or typically lacking peripheral protrusions. Both chloroplasts were slightly diagonally positioned at variable angles to each other, which showed their wide front face and narrow profile (Figs. 2D and 3, B and D). The chloroplasts were in close contact, touching each other at just one point, as shown by confocal microscopy (Fig. 3). This linkage keeps both chloroplasts together, but on an angle, with their opposite ends free. The arrangement was also observed in the aplanospores (Fig. 2, G and H).

### Aplanospores

**Fig. 4:**
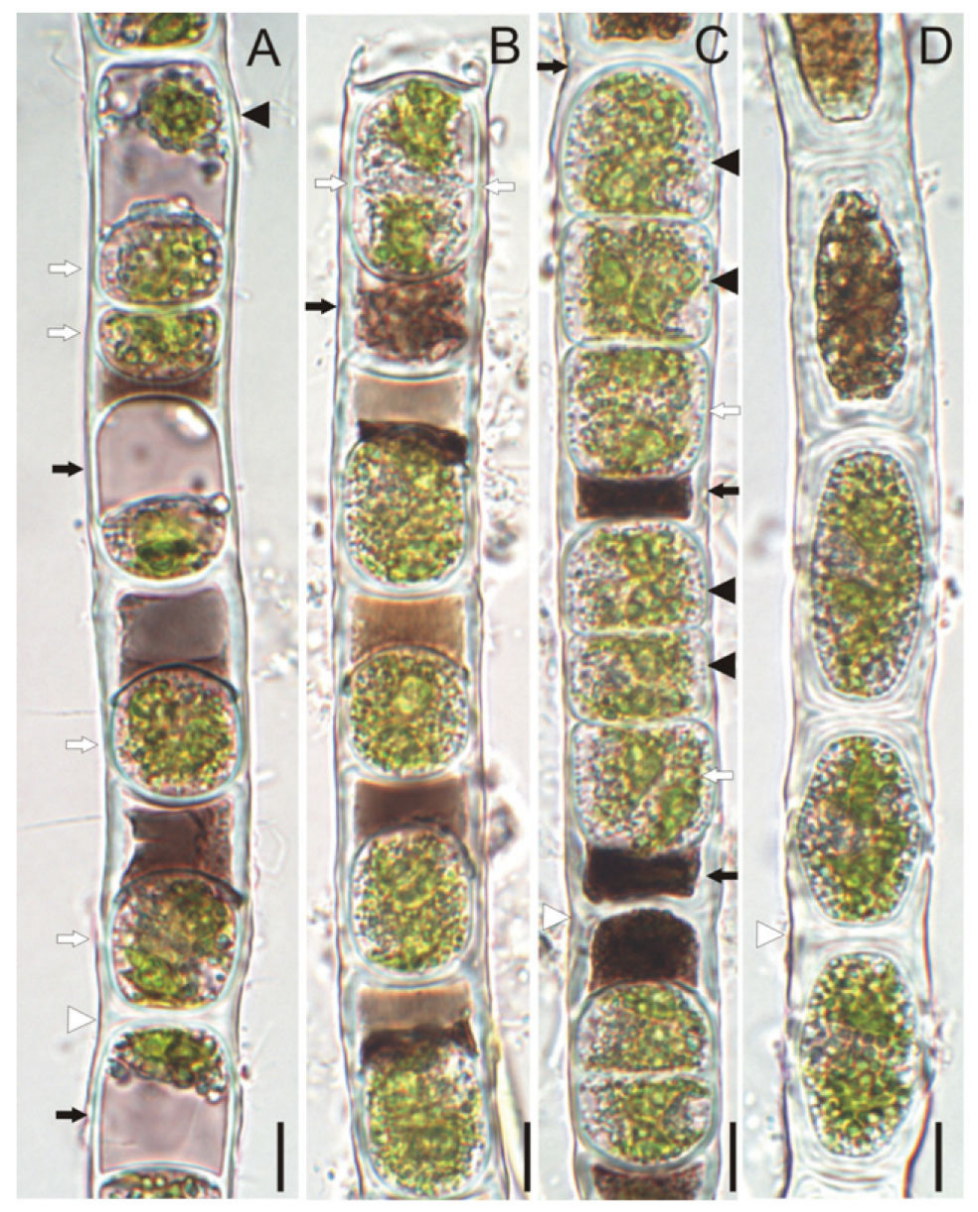
Light microscopic images of reproductive morphology in *Zygogonium ericetorum*: (A) filament in early stages of aplanospore development showing: (i) cells with compacted chloroplasts, nucleus and other cell organelles located near transverse wall in opposite direction to a large vacuole with purple content (black arrows), (ii) completely developed aplanospore (white arrows) separated from a purple-colored vacuole by a colorless wall, (iii) remnant of chloroplast outside aplanospore (black arrowhead), (B) filament in late stages of aplanospore development following the resting period, note enlarged aplanospore with cell wall cleavage (white arrows), and compacted purple-brownish cytoplasmic residue (black arrow), (C) filament with two neighboring aplanospores (white arrows) each germinated into two new vegetative cells (black arrowheads), note very compacted brownish residue (black arrows), (D) akinetes with thick multilayered colorless walls, white arrowhead show H-shaped structures in the pectic layer of cell wall. Scale bars: 10 μm.

**Fig. 5:**
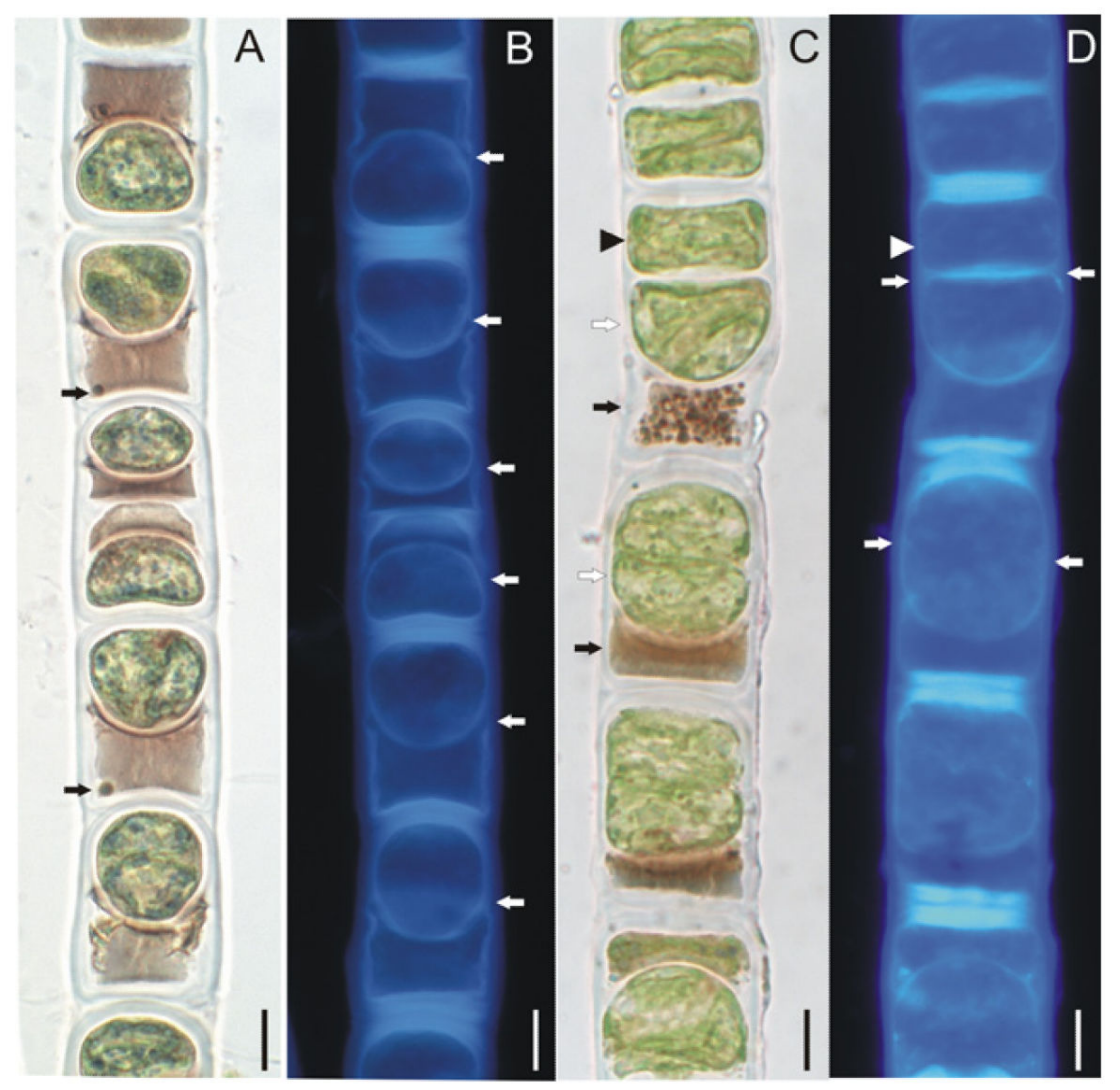
Light (A, C) and fluorescence (B, D) microscopic images of aplanospores in *Zygogonium ericetorum*: (A) filament with completely developed aplanospores, note dark-purple vacuolated cytoplasmic residue with small dense granules (arrows), (B) CFW staining for cellulose, note the ring-like transverse rupture in the cellulose inner layer of vegetative cell wall above the equatorial region of the aplanospore (arrows), (C) Filament with two germinating aplanospores (white arrows) in different stages of cell division and resulting daughter cell (arrowhead), note dense brown cytoplasmic residue (black arrows), (D) CFW staining for cellulose, showing transverse cell wall formation in germinating aplanospores (arrows) and a daughter cell (arrowhead). Filaments CFW and 10% KOH treated. Scale bars: 10 μm.

In purple (surface layer) filaments, numerous vegetative cells formed aplanospores. Their morphology and developmental stages are illustrated in Figures 4 and 5. Aplanospores were globose, ovoid or cylindrical-ovoid, 15–23 μm wide, and 13–28 μm long. They occupied only a portion of the vegetative cell lumen near the transverse wall. Aplanospore development started with compaction of the chloroplasts, nucleus, and other cell organelles, all of which migrated toward the transverse wall in the direction opposite to a large vacuole with purple content (Fig. 4A). Then the aplanospore formed its own smooth, colorless cellulosic wall that separated the chloroplasts and nucleus (Figs. 4, A and B and 5A) from a purple transparent cytoplasmic residue consisting of a large vacuole, and dense, cap-like structure near the aplanospore (Figs. 4, A and B and 5A). Small spherical bodies, and occasionally small chloroplast fragments were visible outside the aplanospore (Figs. 4A and 5A). CFW staining showed a ring-like transverse rupture in the cellulosic inner layer of the vegetative cell wall above the middle area of the aplanospore (Fig. 5B).

Aplanospores did not develop sutures as typically observed in *Zygnema*, and their chloroplasts remained intact during the resting period, a minimum of 30 d. Longitudinal and transverse cell walls of filaments thickened with the aging of aplanospores, and cytoplasmic residue became more condensed and purple-brownish (Figs. 4B and 5, A-C). We observed aplanospore germination into the 30th day of inoculation of the field material in liquid BBM (Figs. 4C and 5, C and D). Germination began with the enlargement of the aplanospores and compaction of purple-brownish cytoplasmic residue, followed by the transverse cleavage of the aplanospore into two nearly equal halves (Figs. 4, B and C and 5, C and D). CFW staining showed that the aplanospore and its daughter cell were enclosed in thick transverse walls of original vegetative cell, and in thin longitudinal walls formed de novo in the area of the ring-like rupture around the aplanospore (Fig. 5D). In this way, each aplanospore formed a short filament of 3–4 new green cells within the sporangium, separated by highly reduced, dense, darkbrown cytoplasmic residues (Fig. 4C). These short few-celled fragments eventually escaped, growing into new filaments. Rarely did the aplanospore, after its enlargement and cell division, break down the sporangium wall to form a laterally growing filament.

### TEM of vegetative cells and aplanospores

**Fig. 6:**
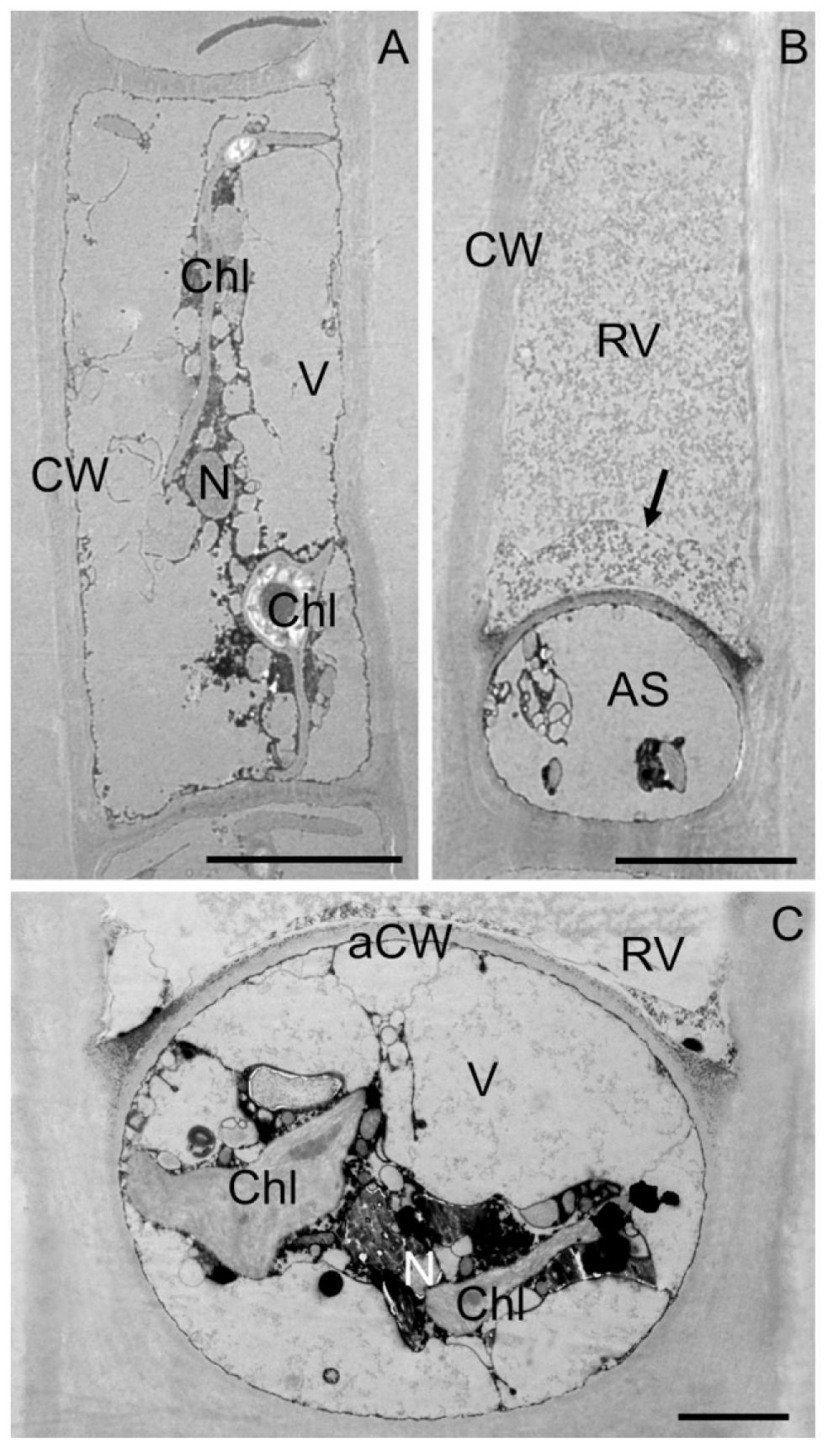
Transmission electron micrographs of longitudinally sectioned vegetative cell (A) and aplanospores (B, C) in *Zygogonium ericetorum*: (A) vegetative cell with a central nucleus, and two chloroplasts in the central part of the cell, (B) cell with aplanospore, note residual vacuole is divided into two parts, electron dense granular structure close to the aplanospore separated by a membrane (arrow), and less electron dense area in the distant part, (C) median section through aplanospore, membrane surrounded residual vacuole outside the aplanospore, aplanospore cell wall with precipitations on the outside, two chloroplasts, nucleus and vacuoles clearly visible. AS, aplanospore; aCW aplanospore cell wall; Chl, chloroplast; CW, cell wall; N, nucleus; RV, residual vacuole; V, vacuole. Scale bars: (A and B) 10 μm, (C) 2 μm.

Vegetative cells and aplanospores were investigated by TEM (Fig. 6). Vegetative cells contained large, electron translucent vacuoles, that covered the entire surface of the cell and only a small central cytoplasmic portion was evident (Fig. 6A). The cytoplasmic portion facing the cell walls was extremely thin (~50 nm), but clearly visible (Fig. 6A). The chloroplasts contained a central, pillow-shaped pyrenoid with clearly visible starch grains (Figs. 6A and S3, A–B in the Supporting Information). In cross-sections, the flat parts of the chloroplast appeared as thin lamina up to 300–400 nm thick (Figs. 6A and S3A), containing about 15 parallel thylakoids, each with a diameter of 15–16 nm (Fig. S3C). In the vicinity of the pyrenoid, plastoglobules with a diameter of ~140 to 220 nm were situated (Fig. S3, A, B, and D).

Aplanospores occupied nearly one-third of the original cell (Fig. 6B) and were covered by a smooth, ~250 nm-thick cell wall (Fig. 6, B and C). Precipitates were visible on the outer surface of the aplanospore wall and in the inner corners of the original cell wall (Fig. 6, B and C). The aplanospores contained the same organelles as the vegetative cells. The vacuoles of the aplanospores appeared electron translucent (Fig. 6, B and C). The chloroplasts had pyrenoids and plastoglobules (Figs. 6C and S3E). In the cellular space outside the aplanospores wall, a membrane-enclosed vacuole was visible (Fig. 6B). The granular structure of this vacuole had varying electron densities, while the region close to the aplanospore was more electron dense, the region distant from the aplanospore was less electron dense. These two regions were separated by a membrane (Fig. 6B). Occasionally, irregular membranous structures (Fig. 6C) or disintegrated parts of chloroplasts were observed outside the aplanospores.

## Discussion

### *Zygogonium ericetorum* phylogeny

There has been considerable disagreement with regard to the taxonomic status of *Zygogonium*. Czurda (1932) considered *Z. ericetorum* to be a species of *Zygnema* and several subsequent authors followed his treatment. Transeau (1933) circumscribed *Zygogonium* more completely and considered six species to belong to the genus, four of which were new combinations transferred from *Zygnema*. However, Stancheva et al. (2012) discovered species with most of the vegetative and reproductive characteristics described by Transeau (1933, 1951) for *Zygogonium* to be phylogenetically embedded in *Zygnema*. Previously, only one strain of *Zygogonium* was available for study, *Z. tunetanum*, from a lake in Canada (not the type locality for the species). We were unable to confirm the identity of this strain because of a lack of reproductive characteristics, and thus the taxonomic status of *Zygogonium* remained in question (Hall et al. 2008, Stancheva et al. 2012). Moreover, the vegetative characteristics of *Z. tunetanum* showed it was more consistent with *Zygnema* than *Zygogonium* (Fig. S4 in the Supporting Information).

This study on the type species of *Zygogonium* showed that the samples of *Z. ericetorum* were difficult to place phylogenetically. In all analyses, *Z. ericetorum* was most closely related to *Mesotaenium* sp. JH0031. However, these two species are very distantly related (i.e., connected by long branches). What is clear is that *Z. ericetorum* is phylogenetically distantly related to other zygnematophytes in terms of sequence divergence of the three genes sampled, including the core group of Zygnematales (Fig. 1). *Zygogonium ericetorum* is also distantly related to the dubiously identified *Z. tunetanum*, and the *Zygnema* clade (Fig. 1). We conclude that this strain of *Z. tunetanum* should, unequivocally, be considered as member of *Zygnema*. Although the phylogenetic placement of *Z. ericetorum* remains uncertain, the species (and consequently the genus *Zygogonium*) is phylogenetically distinct from *Zygnema*.

### Vegetative characters relevant to generic concepts of *Zygogonium* and *Zygnema*

In this study, we assessed the value of morphological features of *Z. ericetorum* previously considered taxonomically important. Many of these characteristics are ambiguously and inaccurately described in the literature (see discussion below), and thus poorly understood. We applied a variety of LM, confocal, cytochemical and TEM techniques, which allowed us to provide a more thorough description of the extremely variable chloroplast shape as well as to provide new structural data on the aplanospores of *Z. ericetorum.*

Our morphological observations on *Z. ericetorum* are in agreement with the existing drawings of its vegetative and reproductive properties in the literature (West and Starkey 1915, Fritsch 1916, Hodgetts 1918, Skuja 1932, Transeau 1933, Randhawa 1959, Tiffany and Britton 1971), and with the LM and TEM visualization of sterile filaments (Hoppert et al. 2004, Lewis and Entwisle 2007, Holzinger et al. 2010, Aigner et al. 2013). This species was originally described as *Conferva ericetorum* by Bory de Saint-Vincent (1797) from the soil of a moor in St. Magne, south-western France, and then illustrated by him as *Leda ericetorum* (Bory de Saint-Vincent 1822a, fig. 8, B–E). Specimens of *Z. ericetorum* from Austria studied here corresponded well with original descriptions and illustrations by Bory de Saint-Vincent (1797, 1822a), which are somewhat vague (see comments below). Confusion in *Z. ericetorum* nomenclature is partially due to Kützing (1843), who proposed a new genus *Zygogonium*, referencing a specimen in his exsiccatae (Kützing Alg. Dec. No. 51). In the exsiccatae, Kützing lists the author of *C. ericetorum* as Roth with no reference to Bory de Saint-Vincent (Kützing’s Exsiccate viewed at the Academy of Natural Sciences, Philadelphia). Roth (1800) provided a Latin description of *C. erictorum*, but when Bory de Saint-Vincent (1822b) transferred *C. ericetorum* to *Leda*, he stated that “our *C. ericetorum* adopted by Roth is probably one of the species.” Because *C. ericetorum* Roth is a later homonym of *C. ericetorum* Bory de Saint-Vincent, Guiry (2013) concluded that *C. ericetorum* Roth was illegitimate and that Kützing should be listed as the author of *Z. ericetorum* and we follow his suggestion. Regardless of the nomenclatural status of *Z. ericetorum*, this is likely the same species as *C. ericetorum* Bory and *C. ericetorum* Roth (illegitimate) (Guiry 2013).

Several authors commented on the occurrence in *Z. ericetorum* of thick, multilayered cell walls with H-shaped structures in response to desiccation (West and Starkey 1915, Fritsch 1916, Transeau 1933, Hoppert et al. 2004, Lewis and Entwisle 2007, Holzinger et al. 2010, Aigner et al. 2013). However, similar multilayered cell walls have been observed in some species of *Zygnema* (Stancheva et al. 2012) and *Mougeotia* (Gauthier-Lièvre 1965) distributed in arid conditions. Rundina (1998) attributed the formation of H-shaped structures in the pectic layer of the cell wall in some *Zygogonium, Zygnema, Mougeotia*, and *Spirogyra* species to their terrestrial habitat and desiccation events with subsequent rewetting of the filaments, which cause rupture of cell walls. Therefore, the cell wall specifics in *Z. ericetorum* are of little taxonomic value, but most likely indicative for the environmental conditions in the habitat, where desiccation events are common (Holzinger et al. 2010).

The chloroplast structure was one of the out-standing cellular characteristics of *Z. ericetorum* and considered to have a high taxonomic value for distinguishing *Zygogonium* from the other members of Zygnematophyceae. Bory de Saint-Vincent (1822a) on figure 8, B–E provided drawings of three filaments with several vegetative cells, each containing chloroplasts with variable shape, ranging from two prominent rounded plates to elongated narrow bands, very closely placed and visible as a single structure. Some early authors (West and Starkey 1915, Fritsch 1916) expressed doubts about the number of chloroplasts per cell, reporting a single chloroplast with one or two pyrenoids in shorter cells of *Z. ericetorum*. However, using a regular LM, both chloroplasts could be hardly visible as two distinct structures connected by an “exceedingly delicate bridge,” as observed only in the long cells by Fritsch (1916). Transeau (1933) characterized the chloroplasts of *Zygogonium* as two rounded plate-like or irregular pillow-shaped, and many phycologists accepted this view (Randhawa 1959, Gauthier-Lièvre 1965, Tiffany and Britton 1971, Bourrelly 1990, Rundina 1998, Johnson 2002, Lewis and Entwisle 2007).

In our observations, the chloroplasts in *Z. ericetorum* were determined to be two per cell of an irregular, compressed shape, which was clearly illustrated by fluorescence LM, CLSM and TEM images of chloroplasts placed at an angle in a cell which displaced their wide front face and narrow profile (Figs. 2D; 3, B and D; and 6A and S2). This understanding corresponds to Bory de Saint-Vincent’s (1822a) illustration of chloroplasts as either large rounded structures, or very narrow bands, clearly indicating their different front and side view as a plate. Similar paired, disc-shaped, variously orientated plastids are characteristic of another genus, *Pleurodiscus* Lagerheim (Smith 1950, Transeau 1951, Randhawa 1959, Bourrelly 1990), considered closely related to *Zygogonium* (Skuja 1932, Yamagishi 1963).

This finding is in contrast to the radial symmetry of the chloroplasts in *Zygnema*, nevertheless some authors considered *Zygnema* and *Zygogonium* having similar stellate chloroplasts (Kadlubowska 1984, Gontcharov et al. 2003). Because of the variable morphology of the chloroplasts in *Z. ericetorum*, which might also be the case for other members of the genus, many filaments in different life stages need to be analyzed to evaluate the correct chloroplast shape in *Zygogonium* and to distinguish the genus from *Zygnema* using regular LM. Currently, the genus *Zygogonium* contains over 30 species (Guiry and Guiry 2014), many of which have chloroplasts characteristic of *Zygnema* (see introduction for example), and therefore the latter genus needs careful taxonomic revision.

### Reproductive characters relevant to generic concepts of *Zygogonium* and *Zygnema*

Taxonomically, reproductive characteristics are very important for defining groups of filamentous Zygnematophyceae. Unfortunately, we were not able to observe sexual reproduction in *Z. erictorum*, but according to Transeau (1951) sexual and asexual reproductive structural characters (i.e., gametangial and sporangial features, and mesospore color and ornamentation) are consistent within the same species. In the studied material, aplanospore formation was a common mode of reproduction. However, we did not observe a sporangial wall dividing the aplanospore from the rest of the cell, which is considered by Transeau (1933) as distinguishing characteristic of *Zygogonium*. On the other hand, the aplanospores and zygospores in *Zygnema aplanosporum* Stancheva, J. D. Hall et Sheath, illustrated by Stancheva et al. (2012), were separated from the sporangium by a sporangial wall, which is a stable character observed in all populations collected from a large geographic area (R. Stancheva, personal observations). Therefore, this sporangial wall is not unique to *Zygogonium*, as a similar structure has been observed in some species of *Zygnema* (Stancheva et al. 2012) and *Mougeotia* (Yamagishi 1963). In fact, our observations suggest that it may not occur in *Z. ericetorum*.

However, the peculiar development of the aplanospore may be characteristic of *Z. ericetorum*. We observed that aplanospores germinated after a 30 d resting period into new vegetative cells by direct cell division. Each aplanospore produced a few new cells within the old filament along with almost complete resorption of the purple residue. Our view is in complete accordance with Fritsch (1916) who described in great detail the formation of daughter-cells by cell-division of the aplanospore where a septum divided the aplanospore into two approximately equal halves. Fritsch (1916) illustrated groups of four cells, produced from a single aplanospore (Fig. 2, G and H), similar to our Figure 4C. West and Starkey (1915) provided an illustration of similar process of aplanospores germination (Fig. 4F), but the authors stated that aplanospores may escape from the filament by breaking through the mother-cell wall. The germination of aplanospores had been recorded approximately a month after their formation (West and Starkey 1915), or immediately with a return of moisture (Fritsch 1916). Fritsch (1916) concluded “the response to drought is thus of very simple kind.” Fritsch (1916) used the term “akinete” for the structure we interpret to be an aplanospore. The aplanospores in *Z. ericetorum* are very simple, compared to typical aplanospores in Zygnematophyceae (for example in *Z. aplanosporum*, LM and SEM illustrated by Stancheva et al. 2012). Aplanospores of *Z. ericetorum* differ from those of most filamentous zygnematophytes in that the wall is not multilayered, but it is colorless, unornamented and lacks an obvious suture. Besides the structural characteristics of the aplanospore, the developmental process by which the spores are formed is unique to this organism. Tiffany and Britton (1971) illustrated the aplanospore germination process in *Z. ericetorum*, where the new germling filament developed through the opening of the aplanospore wall, similar to aplanospore germination in *Zygnema*, but we cannot confirm that observation.

The presence of cytoplasmic residue left in the gametangia after zygospore (and aplanospore) formation is the third diagnostic characteristic of *Zygogonium* proposed by Transeau (1933). Our data showed that only a part of protoplast was used in aplanospore formation in *Z. ericetorum*, and thus a purple cytoplasmic residue was left in the mother cell (sporangium), outside the aplanospore. These observations of aplanospore structure and associated purple residue agreed well with detailed descriptions and illustrations by West and Starkey (1915), Fritsch (1916), Transeau (1933), as well as with more recent authors (Transeau 1951, Randhawa 1959, Tiffany and Britton 1971, Rundina 1998, Lewis and Entwisle 2007, Johnson 2002). Our TEM images showed new details of the aplanospore cell wall, a cap-like structure near the aplanospore and the ultrastructure of the cytoplasmic residue. To our knowledge, aplanospores and the cytoplasmic residue have been studied in *Z. ericetorum* for the first time by TEM. While the aplanospores contain all organelles also found in the vegetative cell, the cytoplasmic residue consists of a large vacuole with granular content of varying electron density. The granular material is tightly packed against the newly formed aplanospore wall. Moreover, the area outside the aplanospores included small granules and rarely chloroplast fragments, but no intact organelles.

Yamagishi (1963) considered the presence of cytoplasmic residue in gametangia (and sporangia) and sporangial wall surrounding the spore as important taxonomic features in defining genera of Zygnematophyceae. According to his concept, conjugation in *Mougeotia* C. Agardh and *Zygogonium* are similar, because the zygospore is surrounded by a sporangial wall separating it from the gametangial cells, and cytoplasmic residue is left in gametangia. However, the structure and composition of cytoplasmic residue is also a character that differs among these genera. For instance in *Mougeotia, Mougeotiella* Yamagishi and *Neozygnema* Yamagishi described a granular appearance or irregularly shrunken membranous structure (Yamagishi 1963, Rundina 1998), while in *Zygnemopsis* (Skuja) Transeau and *Debarya* Wittr. (syn. *Transeauina* Guiry) the lumen of the gametangia outside the spore is filled completely with a bluish-white, refractive pectic or cellulosic layer as the protoplast contracts to the middle of the cell (Transeau 1951). Therefore, we confirm the taxonomic value of the presence and characteristics of cytoplasmic residue in *Zygogonium*, which is a strong diagnostic feature by which to distinguish *Zygogonium* from *Zygnema*.

### Possible function of purple pigmentation and aplanospore formation

The Zygnematophyceae are known to synthesize phenolic compounds (e.g., Han et al. 2007), which might be adaptive in harsh environments. For example galloyl glucose derivatives were described in *Spirogyra varians* (Hassall) Kützing (Nishizawa et al. 1985, Cannell et al. 1988). More recently, unusual phenolic compounds have been described in different arctic and Antarctic *Zygnema* spp. (Pichrtová et al. 2013). Purple to brownish pigmentation of vacuoles has been reported repeatedly in Zygnematophyceae living in extreme habitats like bare ice (e.g., in *Mesotaenium berggrenii* (Wittrock) Lagerheim or *Ancylonema nordenskiöldii* Berggern Remias et al. 2009, 2012b) and attributed to galloylglucopyranose (Remias et al. 2012a).

Evidence suggests that this pigmented residue might be adaptive in environments of high light. In *Z. ericetorum*, the purple pigmentation may have UV shading and antioxidative capacities due to high amounts of soluble phenolic compounds identified (Holzinger et al. 2010, Aigner et al. 2013). Alston (1958) proposed that the purple pigment is an irontannin chemical compound. Recently, Newsome and van Breemen (2012) suggested that it is a highly branched polymer of glucose, containing ester linked polyphenolic moieties such as gallic acid, complexed by ferric iron.

At the present stage, we can only speculate about the importance of aplanospores and cytoplasmic residue in vegetative propagation of *Z. erictorum*. Besides their function as reproductive and resting structures providing protection during unfavorable environmental conditions (Fritsch 1916), they might serve in biochemical cycles in the cell as well. The cytoplasmic residue (i.e., the material that is excluded from the aplanospore) is visibly dark purple or brownish. We hypothesize that this material is excreted from the newly formed aplanospores for protective purposes. The above described chemical composition of the purple pigmentation may be beneficial to the algal cells through antioxidative properties; however such pigmented residues can also be toxic when occurring in higher concentrations, in which case they are stored in vacuoles (e.g., Takahama 2004). The newly formed aplanospores of *Zygogonium* do not have a purple appearance and the new filaments formed from these aplanospores appear mostly green due to the pigments of the larger chloroplast. Under the influence of high UV irradiation occurring under the natural alpine conditions (e.g., Blumthaler 2012), the purple pigmentation is produced again.

## Conclusions

Our results require a careful reconsideration of the taxonomy and ecology of *Zygogonium*. Our collection of *Z. ericetorum* was determined to be distantly related to other zygnematophytes (including *Zygnema* spp.), although confident phylogenetic placement was not possible with our data. Structurally, our specimen of *Z. ericetorum* was largely consistent with its original description and the observations made by subsequent authors (e.g., Fritsch 1916, Transeau 1933) for that species. However, we did not find a sporangial wall separating the aplanospore from the sporangium (as previous authors had suggested) in *Zygogonium*. In contrast, a sporangial wall separated zygospores formed in conjugation tubes, and aplanospores in two *Zygnema* species (Stancheva et al. 2012). Therefore, we conclude that the presence of a sporangial wall should be excluded from the definition of *Zygogonium.* In our observations, *Zygogonium* can be distinguished from other zygnematophytes (particularly *Zygnema*) only when both vegetative and reproductive material is present, based on the combination of two characters: (i) irregular, compressed plate-shaped chloroplasts and (ii) residual cytoplasmic content left in sporangia outside the aplanospore and zygospore. *Z. ericetorum* seems to be extraordinarily well adapted to the terrestrial environment as indicated by its unusual physiology.

### Taxonomic changes

Based on our observations of the type species, *Z. ericetorum*, only a few species are consistent with its peculiar developmental characteristics. Other taxa currently classified as *Zygogonium*, having radial, nearly stellate chloroplasts and lacking cytoplasmic residue after spore formation should be transferred to other genera, most likely *Zygnema*. Many of these species have zygospores with multilayered colored and ornamented mesospores, and are surrounded by a sporangial wall, similar to other *Zygnema* species (Stancheva et al. 2012). Below, we summarize the taxonomic changes necessary to reflect our current understanding of the genus *Zygogonium*. We followed Guiry and Guiry (2014) for the list of *Zygogonium* species currently accepted taxonomically. The lack of cytoplasmic residue in gametangia was confirmed in all 18 species transferred to *Zygnema*, based on the original descriptions (see below). When the chloroplast shape was described it was stellate, except for the irregular sub-globular to globular chloroplasts in *Zygogonium sinense* C.-C. Jao (Jao 1988) and *Z. sphagnophilum* Gauthier-Lièvre (Gauthier-Lièvre 1965).

*Zygnema aquaticum* (Gauthier-Lièvre) Stancheva, J. D. Hall, McCourt et Sheath **comb. nov.**

*Basionym: Zygogonium aquaticum* Gauthier-Lièvre 1965, Beihefte zur Nova Hedwigia 20: p. 87, Pl. 25, figure A a–c.

*Zygnema cyanosphaeroidicum* (O. Bock and W. Bock)

*Synonym*: *Zygogonium cyanosphaeroidicum* (O. Bock and W. Bock) Kadłubowska 1972

*Zygnema exuvielliforme* (C.-C. Jao) Krieger in Kolkwitz and Krieger 1944

Basionym: *Zygogonium exuvielliforme* C.-C. Jao in Taylor 1935

*Zygnema guineense* (Gauthier-Lièvre) Stancheva, J. D. Hall, McCourt et Sheath **comb. nov.**

*Basionym*: *Zygogonium guineense* Gauthier-Lièvre 1965, Beihefte zur Nova Hedwigia 20: p. 89, Pl. 26, figure A.

*Zygnema heydrichii* Schmidle 1897

*Synonym*: *Zygogonium heydrichii* (Schmidle) Transeau 1933

*Zygnema heydrichii* var. *indicum* Randhawa 1938

*Synonym*: *Zygogonium indicum* (Randhawa) Transeau 1951

*Zygnema jaoi* Krieger in Kolkwitz and Krieger 1944

*Synonyms*: *Zygogonium orientale* Y. X. Wei 1979, and *Zygogonium orientale* var. *reniforme* H. J. Hu 1979 (according to Jao 1988), *Zygogonium reniforme* (H. J. Hu) Kadłubowska 1983. Based on *Zygogonium sinense* C.-C. Jao 1935

*Zygnema laetevirens* Klebs 1886

*Synonym: Zygogonium laetevirens* (Klebs) Migula 1907

*Zygnema maghrebianum* (Gauthier-Lièvre) Stancheva, J. D. Hall, McCourt et Sheath **comb. nov.**

*Basionym: Zygogonium maghrebianum* Gauthier-Lièvre 1965. Beihefte zur Nova Hedwigia 20: p. 89, Pl. 27, figure A.

*Zygnema marocanum* (Gauthier-Lièvre) Stancheva, J. D. Hall, McCourt et Sheath **comb. nov.**

*Basionym: Zygogonium marocanum* Gauthier-Lièvre 1965, Beihefte zur Nova Hedwigia 20: p. 90, Pl. 25, figure D.

*Zygnema mayyanadense* (Kothari) Stancheva, J. D. Hall, McCourt et Sheath **comb. nov.**

*Basionym: Zygogonium mayyanadense* Kothari 1971, Phykos 10, p. 106, figs. 1-5.

*Zygnema norvegicum* (Kadłubowska et Langangen) Stancheva, J. D. Hall, McCourt et Sheath **comb. nov.**

Basionym: *Zygogonium norvegicum* Kadłubowska et Langangen 1998, Nova Hedwigia 66: p. 504, figures 1 and 2.

*Zygnema plakountiosporum* (C.-C. Jao) Krieger in Kolkwitz and Krieger 1944

*Basionym: Zygogonium plakountiosporum* C.-C. Jao in Taylor 1935

*Zygnema seuratii* Gauthier-Lièvre 1941

*Synonym: Zygogonium seuratii* (Gauthier-Lièvre) Gauthier-Lièvre 1965

*Zygnema sphagnophilum* (Gauthier-Lièvre) Stancheva, J. D. Hall, McCourt et Sheath **comb. nov.**

*Basionym: Zygogonium sphagnophilum* Gauthier-Lièvre 1965, Beihefte zur Nova Hedwigia 20: p. 91, Pl. 26, figure C a–f.

*Zygnema stephensiae* Transeau in Transeau et al. 1934

*Synonym: Zygogonium stephensiae* (Transeau) Transeau 1951

*Zygnema sudanense* (Gauthier-Lièvre) Stancheva, J. D. Hall, McCourt et Sheath **comb. nov.**

*Basionym: Zygogonium sudanense* Gauthier-Lièvre 1965, Beihefte zur Nova Hedwigia 20: p. 92, Pl. 27, figure D.

*Zygnema tunetanum* (Gauthier-Lièvre) Stancheva, J. D. Hall, McCourt et Sheath **comb. nov.**

*Basionym: Zygogonium tunetanum* Gauthier-Lièvre 1965, Beihefte zur Nova Hedwigia 20: p. 92, Pl. 27, figure E.

## Supplementary Material

S1 Figure S1. Phylogeny of Zygnematophyceae based on a RAxML analysis of *rbcL* showing the relationship between *Zygogonium ericetorum* and a broader sampling of *Mesotaenium* strains. Support values follow Figure 1.

S2 Figure S2. Confocal laser scanning microscopic images of chloroplasts in *Zygogonium ericetorum*, showing their variable morphology: (A–D) sterile vegetative filaments, (E–J) filaments with aplanospores. Scale bars: 10 μm.

S3 Figure S3. Transmission electron micrographs of *Zygogonium ericetorum* chloroplast ultrastructure in vegetative cells (A–D) and aplanospore (E). (A) transverse section through chloroplast, pillow-shaped central part with starch grains clearly visible, flat parts emerging to both sides, numerous plastoglobues (arrow) adjacent to the starch grains, (B) Pyrenoid surrounded by starch grains, plastoglobules (arrow) in the close to starch grains, (C) fine structure of thylakoids in the edge of a chloroplast wing, (D) surface section through central part of chloroplast showing starch grains and numerous plastoglobues, (E) irregular arrangement of thylakoids and plastoglobules in aplanospore. PG, plastoglobules; Py, pyrenoid; S, starch; V, vacuole. Scale bars: (A) 2 μm; (B, D–E) 1 μm; (C) 200 nm.

S4 Figure S4. Light and fluorescence microscopic images of chloroplasts in *Zygogonium tunetanum* UTCC136. Scale bar: 10 μm.

Table S1 Table S1. List of strains used in this study as well as the GenBank number for the sequenced loci. Newly determined sequences are presented in bold typeface.

## Abbreviations

atpB: ATP-synthetase subunit B
BBM: Bold’s Basal Medium
BI: Bayesian inference
CLSM: confocal laser scanning microscopy
ML: maximum likelihood
MP: maximum parsimony
psbC: photosystem II CP43 gene
rbcL: RUBISCO large subunit
TEM: transmission electron microscopy
