# Supplementary figures and images for: PHYLOGENETIC POSITION OF ZYGOGONIUM ERICETORUM (ZYGNEMATOPHYCEAE, CHAROPHYTA) FROM A HIGH ALPINE HABITAT AND ULTRASTRUCTURAL CHARACTERIZATION OF UNUSUAL APLANOSPORES
Source: J Phycol. Author manuscript; Available in PMC 2015 Mar 23. (PMC4370237; doi:10.1111/jpy.12229)

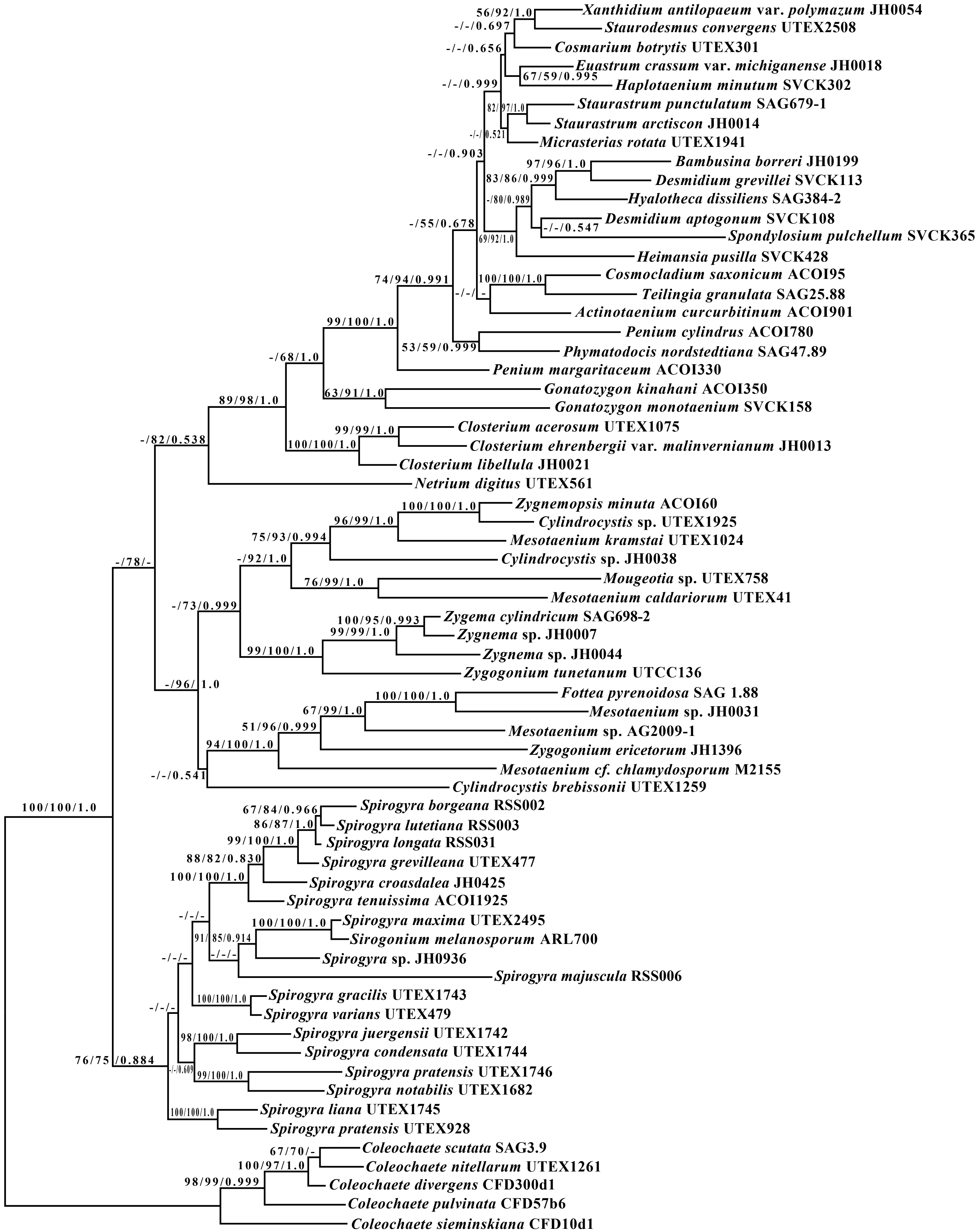

0.07 substitutions/site

Supplement: S1 — Figure S1. Phylogeny of Zygnematophyceae based on a RAxML analysis of rbcL showing the relationship between Zygogonium ericetorum and a broader sampling of Mesotaenium strains. Support values follow Figure 1. [file NIHMS62596-supplement-S1.pdf]

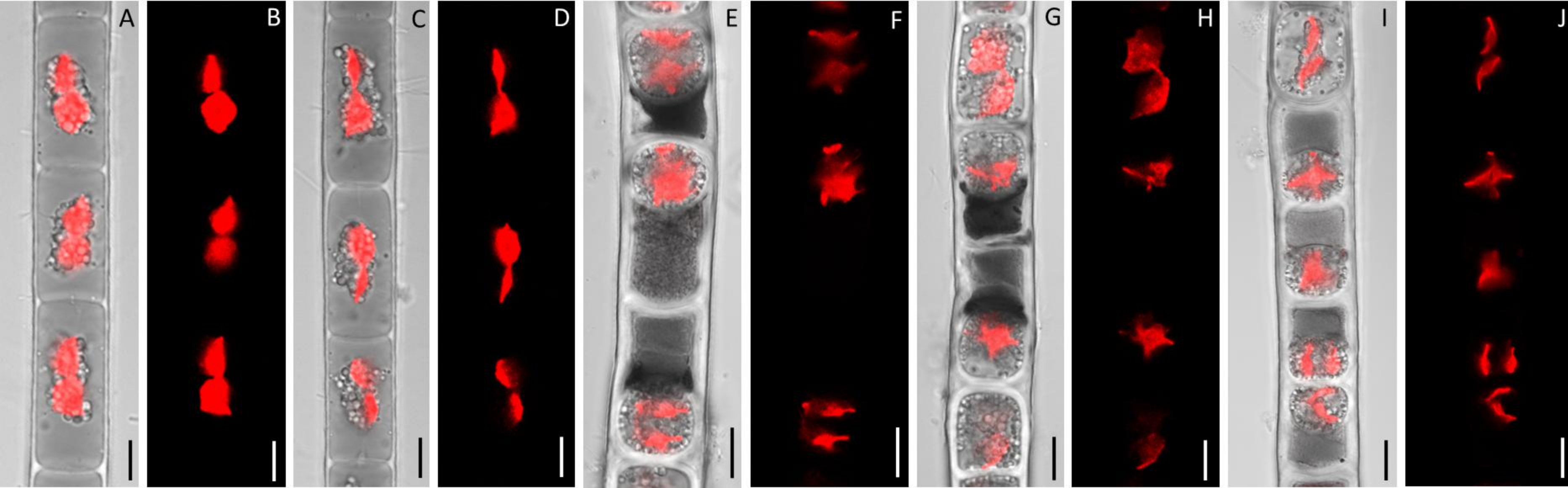

Supplement: S2 — Figure S2. Confocal laser scanning microscopic images of chloroplasts in Zygogonium ericetorum, showing their variable morphology: (A–D) sterile vegetative filaments, (E–J) filaments with aplanospores. Scale bars: 10 μm. [file NIHMS62596-supplement-S2.pdf]

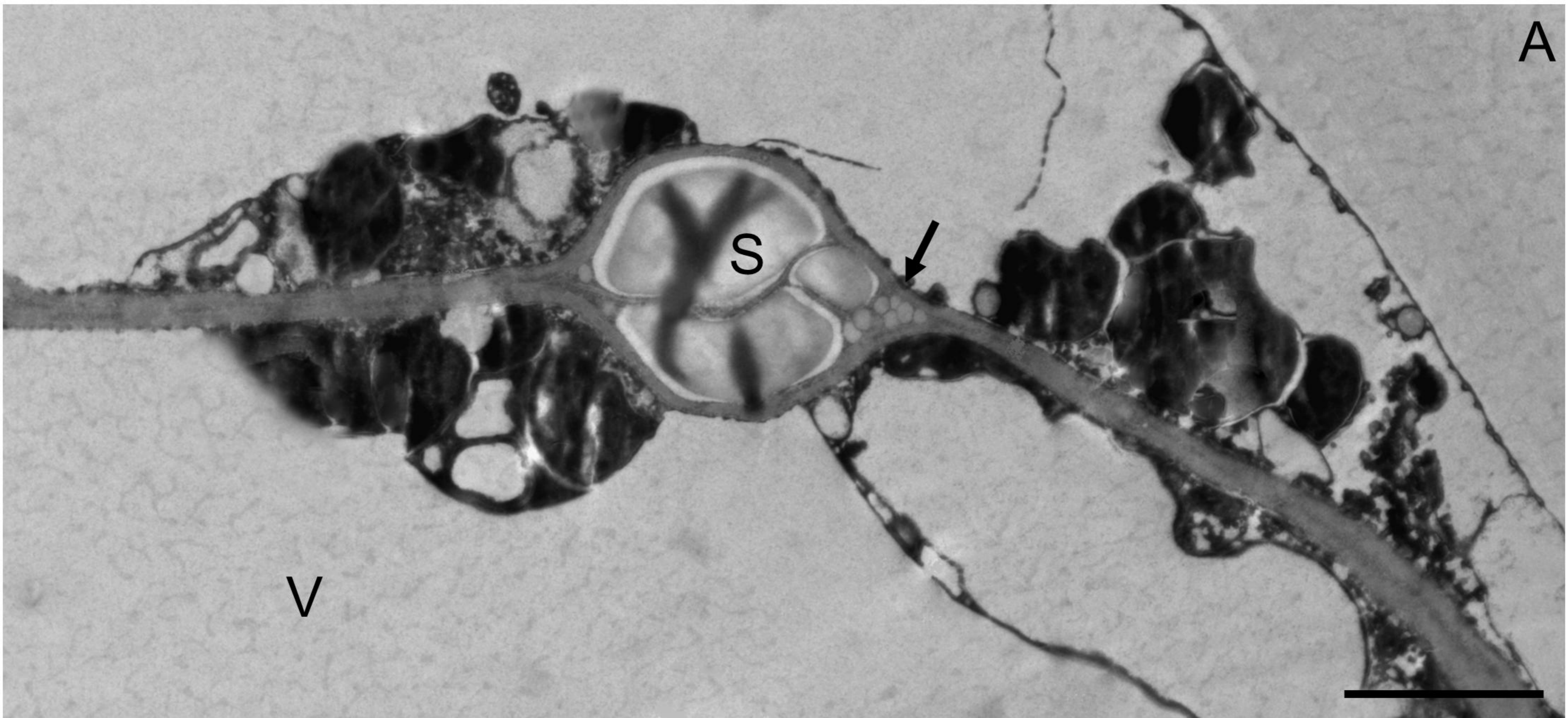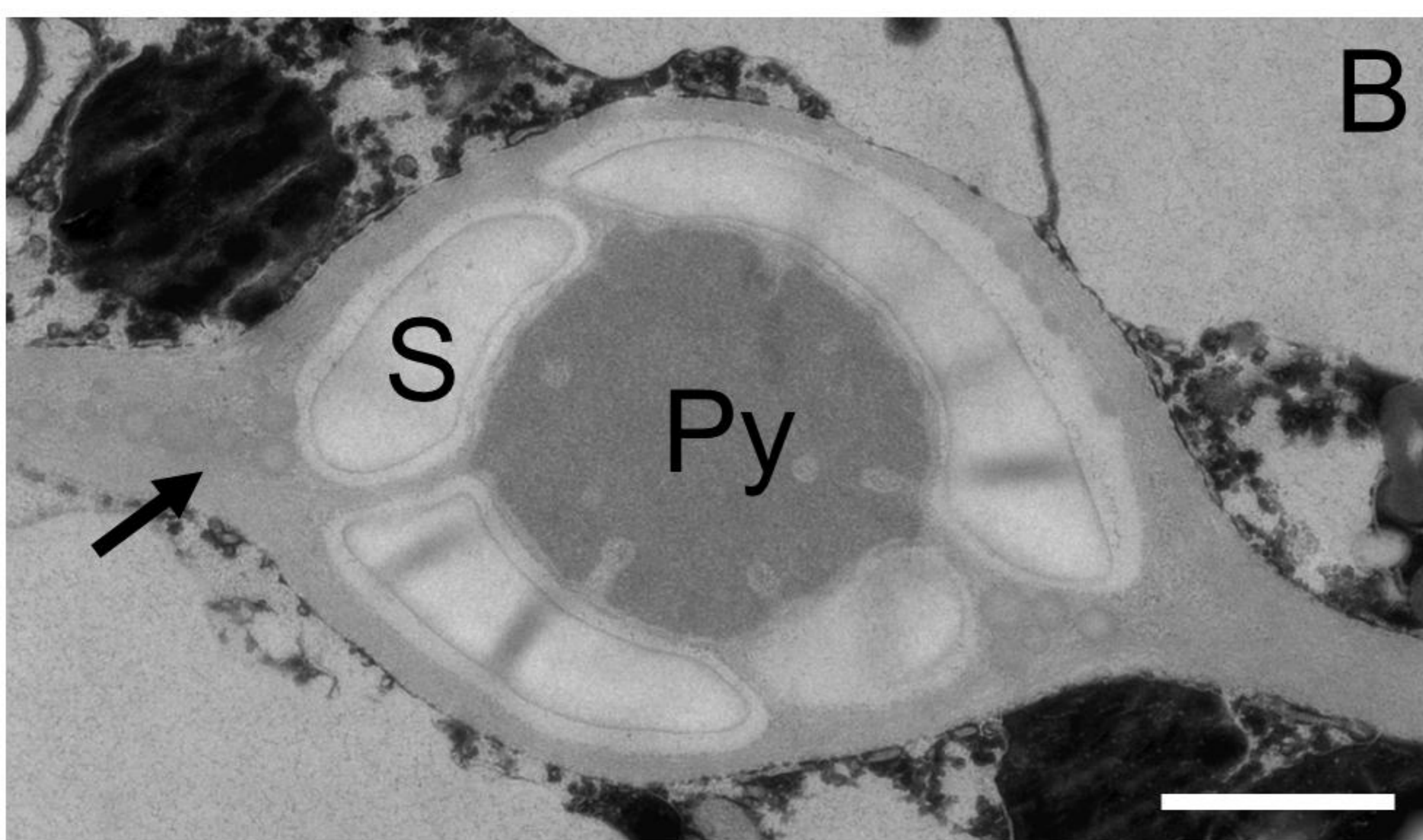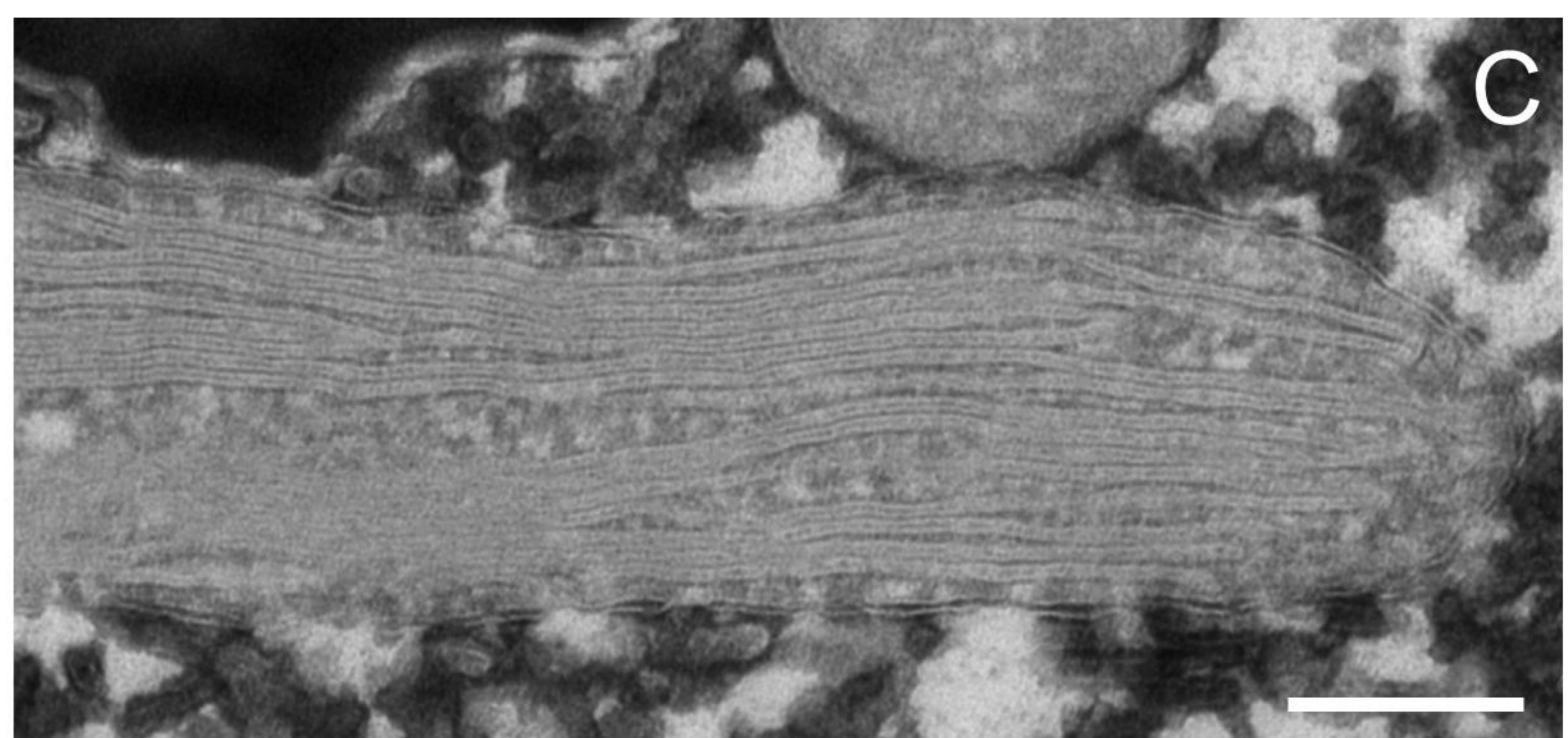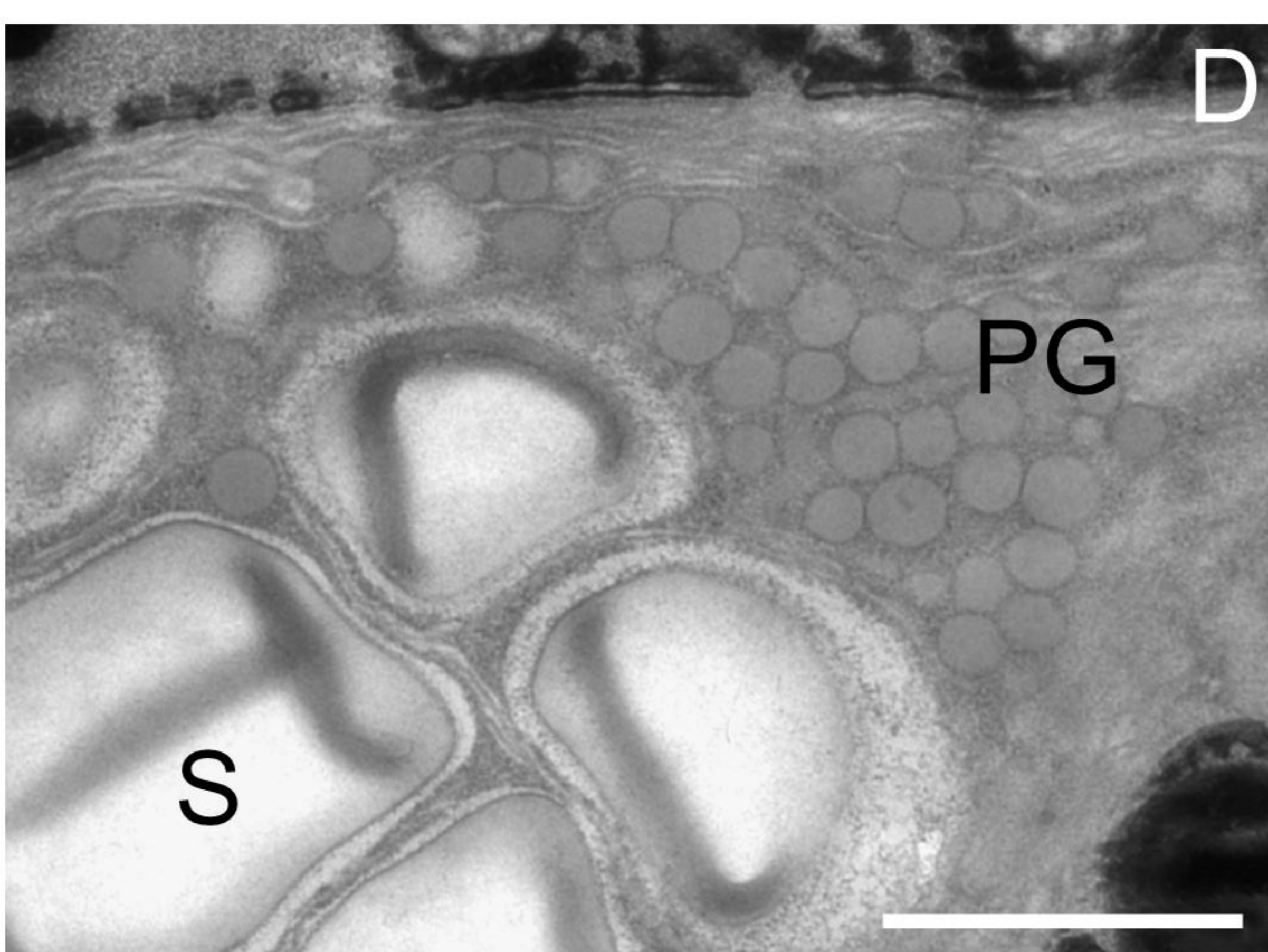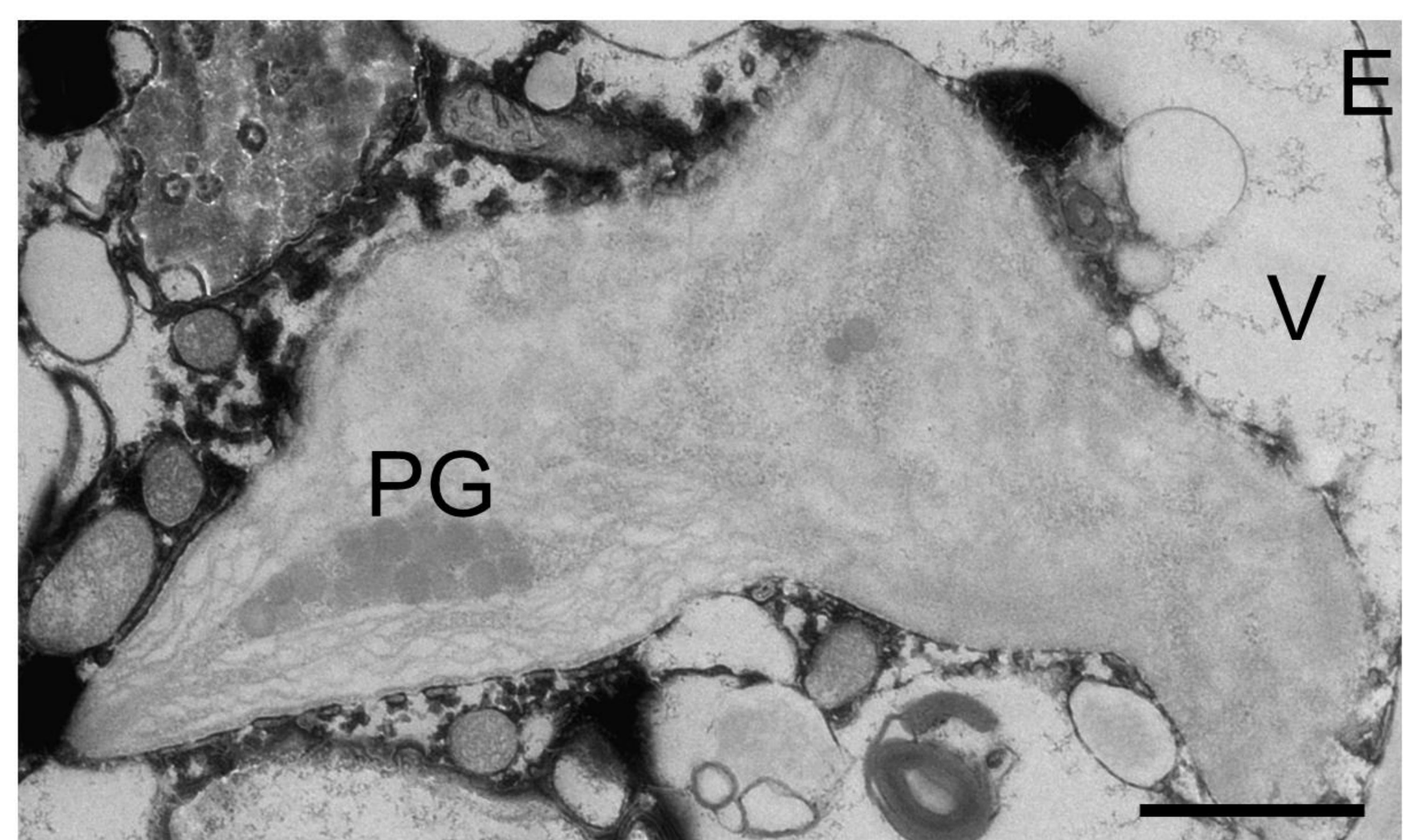

Supplement: S3 — Figure S3. Transmission electron micrographs of Zygogonium ericetorum chloroplast ultrastructure in vegetative cells (A–D) and aplanospore (E). (A) transverse section through chloroplast, pillow-shaped central part with starch grains clearly visible, flat parts emerging to both sides, numerous plastoglobues (arrow) adjacent to the starch grains, (B) Pyrenoid surrounded by starch grains, plastoglobules (arrow) in the close to starch grains, (C) fine structure of thylakoids in the edge of a chloroplast wing, (D) surface section through central part of chloroplast showing starch grains and numerous plastoglobues, (E) irregular arrangement of thylakoids and plastoglobules in aplanospore. PG, plastoglobules; Py, pyrenoid; S, starch; V, vacuole. Scale bars: (A) 2 μm; (B, D–E) 1 μm; (C) 200 nm. [file NIHMS62596-supplement-S3.pdf]

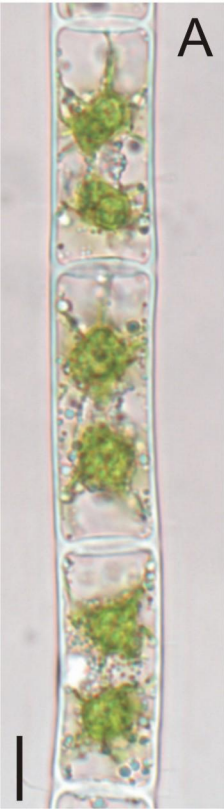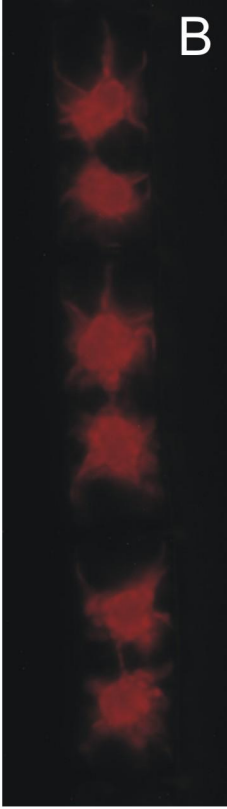

Supplement: S4 — Figure S4. Light and fluorescence microscopic images of chloroplasts in Zygogonium tunetanum UTCC136. Scale bar: 10 μm. [file NIHMS62596-supplement-S4.pdf]
